# Differential Effects of Non-Microbial Biostimulants on Secondary Metabolites and Nitrate Content in Organic Arugula Leaves

**DOI:** 10.3390/foods14142489

**Published:** 2025-07-16

**Authors:** Michele Ciriello, Luana Izzo, Abel Navarré Dopazo, Emanuela Campana, Giuseppe Colla, Giandomenico Corrado, Stefania De Pascale, Youssef Rouphael, Christophe El-Nakhel

**Affiliations:** 1 Department of Agricultural Sciences, University of Naples Federico II, 80055 Portici, Italy; michele.ciriello@unina.it (M.C.); emanuela.campana@unina.it (E.C.); giandomenico.corrado@unina.it (G.C.); depascal@unina.it (S.D.P.); christophe.elnakhel@unina.it (C.E.-N.); 2 Department of Pharmacy, University of Naples Federico II, 80131 Naples, Italy; luana.izzo@unina.it (L.I.); abel.navarredopazo@unina.it (A.N.D.); 3 Department of Agriculture and Forest Sciences, University of Tuscia, 01100 Viterbo, Italy; giucolla@unitus.it

**Keywords:** *Diplotaxis tenuifolia* L., organic farming, non-microbial biostimulants, UHPLC-Q-Orbitrap HRMS analysis, organic acids, glucosinolates, functional food, vegetable protein hydrolyzed

## Abstract

Arugula leaves (*Diplotaxis tenuifolia* L. and *Eruca sativa* L.) are a must-have ingredient in ready-to-eat salads, as they are prized for their appearance, taste, and flavor. The nutraceutical properties of this leafy vegetable are attributed to the presence of valuable secondary metabolites, such as phenolic acids and glucosinolates. Using UHPLC-Q-Orbitrap HRMS analysis and ion chromatography, we characterized the content of phenolic acids, glucosinolates, nitrates, and organic acids in organic arugula [*Diplotaxis tenuifolia* (L.) DC] and evaluated how the foliar application of three different non-microbial biostimulants (a seaweed extract, a vegetable protein hydrolysate, and a tropical plant extract) modulated the expression of these. Although the application of vegetable protein hydrolysate increased, compared to control plants, the nitrate content, the application of the same biostimulant increased the total content of glucosinolates and phenolic acid derivatives by 5.2 and 17.2%. Specifically, the foliar application of the plant-based biostimulant hydrolyzed protein significantly increased the content of glucoerucin (+22.9%), glucocheirolin (+76.8%), and ferulic acid (+94.1%). The highest values of flavonoid derivatives (173.03 μg g^−1^ dw) were recorded from plants subjected to the exogenous application of seaweed extract. The results obtained underscore how biostimulants, depending on their origin and composition, can be exploited not only to improve agronomic performance but also to enhance the nutraceutical content of vegetables, guaranteeing end consumers a product with premium quality characteristics.

## 1. Introduction

The relationship with food accompanies our relationship with life much more than we realize. In fact, Virginia Woolf claimed that “One cannot think well, love well, sleep well, if one has not dined well.” While in the past the concept of “dined well” was attributed to gastronomic pleasure, it is currently linked to the role of food as a psycho-physical regulator. The change in this vision is the result of the new consciousness of today’s consumer. Technology, increased levels of information and education have played a key role in changing consumer preferences and eating habits [1]. The food choices of developed and developing societies have gradually shifted toward increasingly healthy options moving away from previous diets unjustifiably rich in high-calorie, high-fat foods [2]. Not surprisingly, a balanced dietary regimen is often referred to as the mainstay for achieving and maintaining a healthy lifestyle [3].

The scientific community’s unprecedented interest in vegetables with high nutraceutical impact, coupled with consumers’ need to secure a quick, easy, and healthy meal, has contributed to the economic boom in fourth-range vegetable products [4]. The spread of ready-to-use salads, thanks to marketing models that are increasingly efficient in preserving product freshness, have brought arugula (*Eruca sativa* L. and *Diplotaxis tenuifolia* L.) production and consumption back into the limelight [5]. Considering the European market, Italy is currently the leader in leafy vegetable production (lettuce, baby spinach, and arugula), with about 15,000 hectares cultivated mainly in protected crops, distributed between the regions of Campania Veneto and Lombardy [6]. Although arugula was known to the ancient Romans, its use during the imperial age was linked to its aphrodisiac character that made it an indispensable ingredient in love potions [7]. Nowadays, in addition to being used in simple and/or more complex salads, it is used in the production of pesto and is an ever-present ingredient in iconic dishes of Italian cuisine [8,9]. EU Regulation N°. 1258/2011, by introducing stringent and/or specific limitations for nitrate content in arugula (and others), has prompted producers to consider it as the only commodity quality standard. In any case, it should be emphasized that the major concerns toward this anti-nutrient are mainly related to its conversion into harmful by-products primarily nitrites [10]. Unsurprisingly, the impact of nitrates on human health remains uncertain. Pre-clinical and clinical trials did not demonstrated significant correlations between food-borne nitrates and the development of carcinogenic episodes, probably due to the simultaneous intake of specialized metabolites with antioxidant action [11]. As with most *Brassicaceae*, arugula is a valuable source of glucosinolates (GLS). The quantitative–qualitative profile of GLSs typical of arugula, in addition to being primarily responsible for the sought-after flavor of the leaves of this vegetable, defines its superfood character. Regarding specific compounds, arugula has high amounts of glucosativin, glucoerucine, and glucoraphanin. The latter is particularly sought after because of the documented chemoprotective activity exhibited by its breakdown product (sulforaphane) [12,13]. Although, as documented by Pasini et al. [14], analysis of the glucosinolate profile of 37 different arugula accessions did not differ qualitatively, subsequent investigations showed, especially in *D. tenuifolia*, a strong quantitative variability of these nutraceutical compounds underlining the strong genetic imprint. The nutraceutical character of arugula is not limited exclusively to its GLS content. Several studies have shown the presence of significant levels of flavonoids, which being poorly absorbed in the gastro-intestinal tract preserve their antioxidant character all the way to the colon where they would protect epithelial cells from the action of free radicals [15,16]. Long-term consumption of this leafy vegetable, due to the copresence of these two functional secondary metabolites, would ensure a positive impact on human health. However, almost no one consumes enough to explicate these benefits. To address this challenge, one could further promote increased the consumption of leafy vegetables or work to improve the phytochemical density and thus the nutritional quality of the product itself. As has previously been carried out for other *Brassicaceae*, genetic breeding could be the answer to the above, as it would allow for improved phytochemical content [17]. However, specifically for arugula, the limited number of cultivars and the lack of complete sequencing of its genome pose limitations to this strategy. In any case, since phenolic compounds and glycosinolates are actively involved in plant defense, their biosynthesis can be stimulated by abiotic and/or biotic factors or induced indirectly by the use of natural products that activate the plant’s defense mechanisms [18,19]. Although the application of natural biostimulants can have a positive effect on the health potential of plants, most reports have focused on the beneficial effects in terms of yield. The ability to promote plant growth regardless of mineral content has made the use of biostimulants indispensable in organic farming contexts [20]. In recent decades, in response to increasing market demands, the area under organic cultivation has increased significantly. Again, the consumer has played a key role. Compared to conventional products, organic products—due to the reduction in pesticides and heavy metals—are perceived by the consumer as safer and healthier [21]. The well-established positive action of biostimulants on organic and non-organic crops must be accompanied by more studies about their role on nutraceutical quality improvement. Several studies conducted on crops of agronomic interest have shown that exogenous application of plant biostimulants significantly increased the production of specialized metabolites [22,23,24]. Specifically, Gavelienė et al. [25] and Ganugi et al. [26] observed following the application of microbial biostimulants an improvement in the quality characteristics of organically grown carrots and tomatoes. As regards the main non-microbial biostimulants (protein hydrolysates, plant and algal extracts), the biostimulating action is mainly linked to the presence of specific signal molecules (organic molecules, such as amino acids, peptides, carbohydrates, and vitamins) that trigger different gene regulation mechanisms capable of determining phenotypic responses that, in addition to culminating in an improvement in production, can upregulate the production of key specialized metabolites [27].

The present research is a continuation of the work where we investigated the morpho-physiological and productive response of arugula plants subjected to the foliar application of different biostimulants of non-microbial origin [28]. In light of the above-described production results and considering the close relationship between plant biostimulants and secondary metabolites, our research aims to characterize from a phytochemical point of view arugula production in an organic farming context by going to identify, through UHPLC-Q-Orbitrap HRMS analysis and ion chromatography, the potential beneficial effects of three different non-microbial biostimulants.

## 2. Materials and Methods

### 2.1. Experimental Design, Treatments, and Sample Collection

The experimental trial was conducted at the Altamura farm located in Pontecagnano Faiano (Salerno, Italy) during the winter–spring season for a total duration of 36 days. The soil of the experimental site, characterized by a loamy–clayey texture, had total nitrogen (N_tot_), exchangeable potassium, and Olsen phosphorus contents of 0.09%, 191.1, and 105.6 mg kg^−1^, respectively. A green manure with sorghum (*Sorghum bicolor* L.) was planted prior to sowing, followed by solarization to reduce biotic pressure. Irrigation management was carried out through the use of a drip irrigation system, while for pathogen, fungal, and insect management, normal agricultural practices allowed in organic cultivation were carried out. A randomized complete block design was used for the present experiment where treatments were replicated four times. Specifically, three different non-microbial biostimulants were compared to an untreated control on arugula (*Diplotaxis tenuifolia* (L.) DC.) plants. The biostimulants were a plant protein hydrolysate (Trainer^®^ hereafter V-PH), a plant extract (Auxym^®^ hereafter PE), and an algae extract (ABYSS^®^ hereafter SWE) (Supplementary Table S1). Each biostimulant was applied weekly by foliar application at the recommended doses of 3.0, 1.0, and 1.5 mL L^−1^.

The doses used in this experiment, in addition to being in accordance with what was indicated by the manufacturing companies, were also selected considering what was reported in the literature in similar experiments [29]. Each experimental unit was 1 m^2^ in size, respectively. Agricultural practices as well as growth conditions have been reported in detail in the previous work by Ciriello et al. [28]. At the end of the growth cycle for each experimental unit, plant tissue samples were collected and placed immediately in liquid nitrogen. The frozen samples were then placed in the freeze-dryer, and when they reached constant weight (about 72 h), the dry samples were ground and used for the qualitative analyses described later.

### 2.2. Determination of Nitrate and Organic Acid Content

In accordance with the methodology detailed by Formisano et al. [30], nitrate and organic acid (oxalate, malate, and citrate) contents in arugula leaves were determined by ion chromatography (ICS3000, Thermo Scientific™ Dionex™, Sunnyvale, CA, USA). Nitrate concentrations (expressed as mg kg^−1^ FW) as well as organic acids (expressed as g kg^−1^ DW) were quantified by comparing peak areas of the samples with certified reference standards using Chromeleon™ 6.8 Chromatography Data System software (Thermo Scientific™ Dionex™, Sunnyvale, CA, USA).

#### Reagents and Chemicals

Water and methanol LC-MS grade, formic acid 99.98% purity (FA), ammonium acetate, GLSs (glucotropaeolin and sinigrin), and phenolic compounds (quinic acid, protocatechuic acid, chlorogenic acid, caffeic acid, p-coumaric acid, genistein, ferulic acid, naringin, rutin, myricitrin, quercetin, kaempferol, apigenin, luteolin, and quercetin 3-galattoside) standards were supplied by Merck KGaA (Darmstadt, Germany). Standards solutions were prepared in methanol at 1 mg mL^−1^, and calibration curves (0.01 to 5 mg L^−1^) were performed with 0.1% FA water.

### 2.3. Extraction Method

#### 2.3.1. GLSs

Powdered samples (40 mg) were dissolved with 1.125 mL of water:methanol (30:70) and sonicated for 30 min in 15 mL tubes. The tubes were then placed in a bath at 70 °C with agitation (120 rpm) for 10 min, after which the samples were cooled on ice for 1 min. Samples were centrifuged (12,000 rpm, 3 min, 4 °C), and the supernatant was collected, while the resulting pellets were extracted again with the same methodology. The two supernatants were combined and filtered (0.22 µm nylon filter) for further analysis.

#### 2.3.2. Phenolic Compounds

Dry powder samples (100 mg) were dissolved in 5 mL of a methanol: water mixture (60:40) in 15 mL tubes and vortexed for 1 min, after which they were sonicated for 15 min in cold. After sonication, samples were shaken for another 15 min and finally vortexed for 1 min. They were centrifuged (5000 rpm, 5 min, 4 °C), and the supernatant was filtered (0.22 µm) and diluted 1:5 with the water:methanol mixture for further analysis.

#### 2.3.3. UHPLC-Q-Orbitrap HRMS Analysis

The chromatographic separation was performed using a UHPLC system (Thermo Fisher Scientific, Waltham, MA, USA) provided with a quaternary pump, an autosampler (Dionex Ultimate 3000), and a system for degassing. Detection and quantification were conducted by Q-Orbitrap HRMS (Thermo Fisher Scientific, Waltham, MA, USA). Data processing was made using Quan/Qual Browser Xcalibur software, v. 3.1.66.10 (Thermo Fisher Scientific, Waltham, MA, USA) [31].

### 2.4. GLSs

Glucotropaeolin and sinigrin standards were used for the semi-quantification analysis purpose. For GLSs separation, the mobile phases consisted of water (A) and methanol (B), each with 0.1% FA and 0.15 mM ammonium acetate. A gradient was used, with a constant flow rate of 0.4 mL min^−1^, such as up to 0.5 min, 100% A; 1 min 60% A, maintained until 3.3 min; 5.7 min 5% A, maintained until 6 min; 7 min 100% A, maintained until 8 min for re-equilibration of the column. The column used was a Luna Omega (Phenomenex, Torrance, CA, USA) C18 100 Å (50 × 2.1 mm, 1.6 µm) at 25 °C and the injection volume was 5 µL. For detection, the compounds were subjected to an electrospray ionization source in negative ionization mode, and each GLS was identified based on its deprotonated molecular ion ([M–H]^−^). Instrumental parameters were sheath gas flow rate, 30 arbitrary units (au); auxiliary gas flow rate, 10 au; auxiliary gas heater, 300 °C; spray voltage, −3.5 kV; capillary temperature, 320 °C; S-lens RF level, 50. The scan range was set between 80 and 500 *m*/*z*, and two scanning events were established—full ion MS and all ion fragmentation (AIF). The full-scan data were obtained with a resolving power of 70,000 full width at half maximum (FWHM) at 200 *m*/*z*, while the AIF scanning events were acquired with a resolving power of 17,500 FWMH with collision energies of 20 and 30 eV.

### 2.5. Phenolic Compounds

Phenolic compound standards were used for the quantitative analysis, except for quercetin-3-o-feruloyl-sophoros, for which a semi-quantitative analysis was performed using the routine curve. For chromatography, mobile phases used consisted of water (A) and methanol (B) both with 0.1% FA for the chromatographic separation. A gradient was used, with a flow rate of 0.4 mL/min, as follows: up to 1 min, 100% A; from 1 to 3 min, 20% A; 6 min, 0% A; 8 min, 100% A for column re-equilibration. The column used consisted of a Kinetex F5 (Phenomenex, Torrance, CA, USA) 1.7 μm (50 × 2.1 mm) at 25 °C. For mass spectrometry detection, the negative ionization mode was used, identifying the deprotonated molecular ion of each compound, in full ion MS scan mode. Instrument parameters were capillary temperature, 320 °C; auxiliary gas heater temperature, 350 °C; spray voltage, 3.5 kV; S-lens RF level, 60; scan range, between 80 and 1000 *m*/*z*, resolution power of 70,000 FWMH, sheath gas flow rate, 18 au and auxiliary gas flow rate, 3 au.

### 2.6. Data Analysis

All mean effects were subjected to one-way ANOVA analysis. Significant differences that were found between the different treatments analyzed were determined by Tukey’s HSD test. All data are reported as mean ± standard error, n = 4.

## 3. Results and Discussion

It is for all to see how the current world of research has, in recent years, emphasized the positive effects of the application of bioproducts with biostimulant action on the growth, development, and yield of several crops of agronomic interest. However, the application of these bioproducts can also trigger substantial changes on the health potential and nutritional value of plants. The results reported in this study highlight how the application and different origin of biostimulants affect the expression of important quality traits in wild arugula grown in an organic farming context.

### 3.1. Nitrate Content: Influence of Non-Microbial Biostimulants

**Figure 1:**
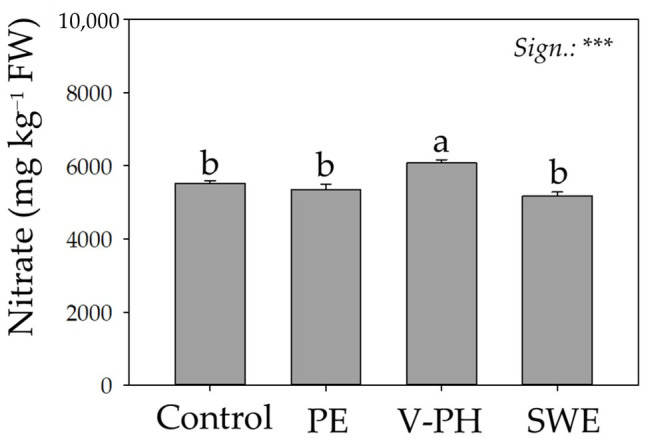
Effect of different non-microbial biostimulants on the nitrate concentration of greenhouse-grown wild arugula. Different letters indicate significant mean differences. ***: *p* ≤ 0.001.

As with most leafy vegetables, wild arugula has the undesirable problem associated with the excessive accumulation of nitrate [32,33]. Recognized and referred to as an anti-nutrient (as a precursor to carcinogenic compounds), the nitrate content has over time become a commodity and nutritional discriminator for arugula and beyond. However, it has recently been proposed that the intake of nitrate-rich foods, especially for athletes, may provide important cardiovascular benefits [34]. Specifically, the consumption of foods characterized by high nitrate content (such as arugula) can increase plasma nitrate and nitrite levels by significantly reducing blood pressure [35]. In any case, in order to be marketed, arugula must have a nitrate content of no more than 6000 mg kg^−1^ (on a fresh basis) during the summer season or 7000 mg kg^−1^ during the winter season (the reference season of our experimentation) (EU Regulation 1258/2011). Factors such as genotype, environmental conditions (as they are directly involved in nitrate reductase expression), and crop management play a key role on nitrate content [36]. In the present study, regardless of the biostimulant treatments evaluated, the nitrate content was below the maximum limits imposed by the European Community for the safe marketing of winter arugula (Figure 1) (EU Regulation 1258/2011).

As shown in Figure 1, compared to the control condition, the different origins of the biostimulants tested in the present experiment distinctly influenced the nitrate content in organic arugula. While the application of the products based on tropical plant extracts (PE) and seaweed (SWE) did not result in significant differences from the nitrate values recorded in the control plants, the use of the biostimulant based on vegetable-derived protein hydrolysates (V-PH) resulted in a 10% increase. We hypothesize that the higher nitrate accumulation in the V-PH-treated arugula plants could be a direct consequence of a more efficient and developed root system (in terms of secondary branching and overall root biomass), which would have increased the rate of nitrate uptake and translocation into the edible portion. In contrast, the application of biostimulants based on seaweed and tropical plant extracts had nitrate values comparable to the control. While the nonvariation of this anti-nutrient is a strength, at the same time, the different result obtained with respect to the V-PH biostimulant underscores how the different origin and composition of biostimulants can affect metabolic pathways differently.

### 3.2. Biostimulant-Specific Enhancement of Glucosinolate Content in Organic Arugula

The identified GLSs were reported in Table 1, including common and systematic names and major ions. Regardless of the application of non-microbial biostimulants, the GLS profiles of organic arugula were very similar. Specifically, the results revealed seven aliphatic-derived compounds (glucoerucin, glucoalyssin, glucoraphenin, glucoiberin, progoitrin, glucoraphanin, glucocheirolin), one indole-derived compound (glucobrassicin), and two aromatic-derived compounds (glucosinalbin and gluconasturtin).

**Figure 2:**
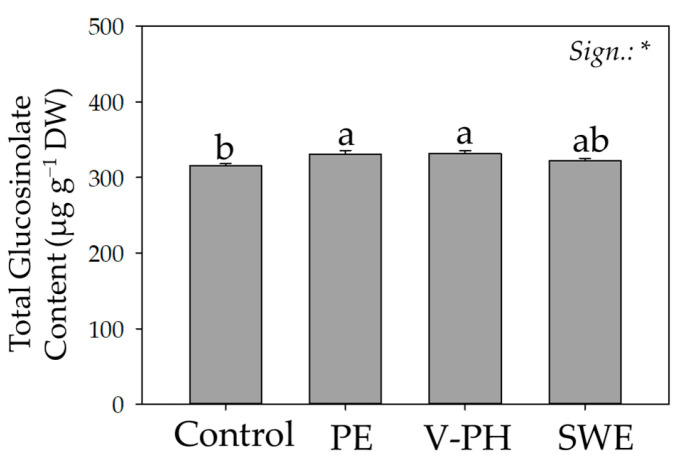
Effect of different non-microbial biostimulants on total glucosinolate content of greenhouse-grown wild arugula. Different letters indicate significant mean differences. *: *p* ≤ 0.05.

The average total content of all identified GLS was 325.12 µg g^−1^ dw and ranged from 315.44 to 331.73 µg g^−1^ dw, recorded from control and V-PH-treated plants, respectively (Figure 2). Consistent with the reports of Pasini et al. [14] aliphatic GLS accounted for about 90% of the total GLS (Table 2). Regardless of biostimulant application, glucoerucine and glucoraphanin were the predominant GLSs, accounting for more than 85 percent of total GLSs (Table 2).

The complexity and quantitative–qualitative ratios of the different GLSs significantly influence the perceived spiciness of arugula leaves [37]. In any case, a detailed review of the literature found that the typical taste of arugula is not directly related to the most abundant GLS [38]. Not surprisingly, glucoraphanin, the most representative component of the GLS profile, would contribute negatively to the typical arugula flavor, while the same authors would argue that progoitrin, although a minority component of the GLS profile, is responsible for the much sought-after bitterness of arugula [38]. Although gluraphanin would contribute negatively to the sensory quality of arugula, sulforaphane, an isothiocyanate derived from glucoraphanin, has recognized bioactive potential because of its anticarcinogenic properties [39]. Its extreme importance to human health would justify the development of products naturally enriched with glucoraphanin; in any case, as shown in Table 2, none of the non-microbial biostimulants tested provided this desired effect. As reported by Xu et al. [40], glucoraphanin would act against adiposity and hepatic steatosis by promoting energy use and preventing lipogenesis and oxidative stress in the liver. On the contrary, compared to control conditions, the application of the biostimulants, regardless of their origin, positively influenced the biosynthesis of glucoerucin (on average +17.5%), a GLS that has been much studied for its bioactivity. This unique response would suggest a similar biostimulation action mediated by the biostimulants of different origin. Although information on the actual chemopreventive potentials of glucoerucin is still scarce, the study by Wagner et al. [41] reports that the main hydrolysis product of this GLS has chemopreventive effects in vivo in C57BL/6 mice and in vitro in HT-29 human cell lines. In addition, a recent study showed that erucin, the main hydrolysis product of glucoerucin, would exert potent protective activity (even at micromolar concentrations) on endothelial dysfunction caused by lipopolysaccharide (LPS)-induced inflammation and conditions typical of diabetes [42]. Compared to the control, the increase in total GLS content recorded in arugula plants treated with PE and V-PH (Figure 2) confirms how the use of natural bioproducts can trigger the activation of specific defensive mechanisms.

This mechanism can be linked to the priming action of biostimulants that would allow the treated plant to react more readily to abiotic stressors through the upregulation of genes related to priming and systemic-acquired resistance (SAR) [43]. In addition to the priming effect, the higher GLS values recorded in PE- and V-PH-treated plants could be a direct consequence of the documented enhancement of nitrogen and carbon metabolic pathways that would provide more usable energy for the biosynthesis of the precursors of the aforementioned bioactive compounds [43,44]. Last but not least, changes related to secondary metabolites could be connected, as supported by Lephatsi et al. [45], to hormonal changes induced by the application of biostimulants, which would lead to an increase in defensive compounds especially in non-optimal growth conditions (as in our case organic cultivation). Specifically, we can speculate that the increased production of secondary metabolites may also be partly attributable to an improvement in the morpho-physiological activities and characteristics of plants treated with PE and V-PH biostimulants as confirmed by the agronomic data presented in the study by Ciriello et al. [28].

### 3.3. Targeted Enhancement of Phenolic Compound Content in Organic Arugula by Biostimulants

Table 3 shows all identified flavonolic and phenolic compounds, including common and systematic names and major ions. A total of four flavonolic compounds (myricitrin, rutin, quercetin-3-o-feruloyyl-sophoros, and quercetin 3 galactoside) and three phenolic acids (chlorogenic acid, caffeic acid, and ferulic acid) were identified.

Regardless of the application of the biostimulants, the flavonol profile of arugula was characterized by a dominant presence of quercetin derivatives, a genetic fingerprint that allows us to distinguish/categorize Diplotaxis accessions from those of Eruca, which are instead characterized by a more abundant presence of Kaempferol aglycones [38,46]. The specific presence of the different aglycones in addition to the premise of distinguishing Diplotaxis accessions from Eruca accessions ensures a different antioxidant capacity. Generally, the anti-inflammatory and antioxidant properties of flavonols are directly related to protecting the colon from oxidative damage caused by free radicals [47]. In any case, the different arrangement of hydroxyl groups and the different degree of glyoxidation significantly influence the antioxidant activity of flavonol compounds [48,49]. This is precisely why quercetin derivatives are characterized by higher antioxidant activity than kaempferol derivatives [50,51]. In contrast to what was previously described for GLS, the application of the non-microbial biostimulants tested resulted in significant augmentative and/or diminutive changes for all phenolic compounds depending on the target molecule (Table 4). As reported in Table 4, compared to the control condition, the foliar application of the biostimulants PE and V-PH reduced the content of chlorogenic acid (on average by 43.9%) and myricitrin (on average by 53.4%), but increased the content of ferulic acid by 69.1% on average. In contrast, the foliar application of SWE increased, compared to the control, the content of ferulic acid, myricitrin, and quercetin 3 galactoside by 60.3%, 45.2%, and 40.6,% respectively.

**Figure 3:**
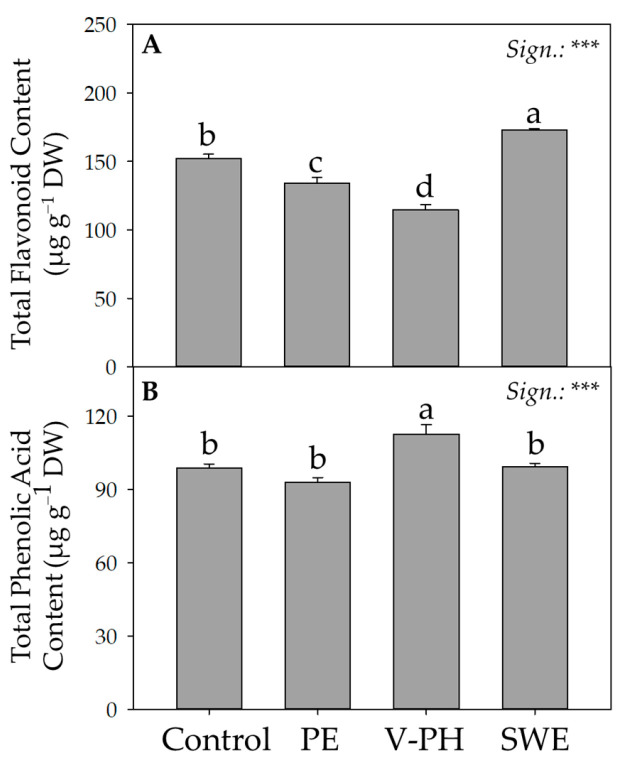
Effect of different non-microbial biostimulants on flavonoid total (**A**) and phenolic acid (**B**) content of greenhouse-grown wild arugula. Different letters indicate significant mean differences. ***: *p* ≤ 0.001.

As shown in Figure 3A, the average total content of all identified flavonol compounds was 143.5 μg g^−1^ dw and ranged from 114.8 to 173.0 μg g^−1^ dw, recorded in V-PH- and SWE-treated plants, respectively. The significant increase (+13.5%) in total flavonol contents recorded in SWE-treated arugula plants compared to control plants confirms the results previously described by Flores et al. [52] and Ali et al. [53]. As suggested by the same authors, the exogenous application of the seaweed extracts would increase the flavonol content due to a significant increase in enzyme activity related to plant defense (ammonia lyase, peroxidase, phenylalanine, polyphenol oxidase, β-1,3-glucanase, and chitinase). Since arugula does not require cooking, and consequently, there is low or no thermal degradation and/or degradation by myrosinase, a significant increase in flavonols would ensure greater effectiveness of the already mentioned and known beneficial effects in vivo [34]. In sharp contrast to what was observed for flavonols, the highest values of total phenolic acids (Figure 3B) were recorded in arugula plants treated with V-PH. These contrasting differences highlight how the varying origin of non-microbial biostimulants promotes distinct metabolic profiles, suggesting the biostimulant-specific activation of different mechanisms of action involved in the expression of secondary metabolites. In light of the contrasting results recorded for the two different phenolic classes analyzed, the choice of the ‘best’ treatment should be made considering the different bioactivity of the compounds involved and how they can influence consumer perception.

Defining the cause-and-effect relationships between the application of the different biostimulants tested and the specific enhancement of the several classes of secondary metabolites analyzed is greatly complicated by the presence in each biostimulant of complex organic multicomponents that synergistically act on the primary and secondary metabolic pathways of plants. However, as reported in a recent review of the literature on non-microbial biostimulants, the exogenous application of V-PH, in addition to increasing the expression of genes encoding for oxidative and reductive carbon metabolism (fumarate dehydrogenase, malate dehydrogenase, phosphoenolpyruvate carboxylase, and phosphoenolpyruvate carboxykinase, RuBisCo) and for nitrogen metabolism (nitrate reductase, glutamine synthase, glutamine-dependent asparagine synthase, and aspartate aminotransferase) would seem to be directly involved in the overexpression of the phenylalanine ammonia lyase (PAL) gene directly involved in the biosynthesis of phenolic compounds [54]. This would confirm the higher concentrations of phenolic acids recorded in the VP-H biostimulant treatments (Figure 3B).

Regardless of the application of the biostimulants, the most abundant phenolic acid identified in arugula plant tissues was found to be caffeic acid (Table 4). In any case, the positive effects of foliar application of V-PH mainly concerned the ferulic acid content (Table 4). Unlike other phenolic acids, ferulic acid was found to be readily absorbed throughout the gastrointestinal tract and metabolized mainly in the liver [55]. A tomato study reported that only 10–25% of ferulic acid was excreted in urine by humans, emphasizing that this phenolic acid is efficiently absorbed from the diet [56].

### 3.4. Organic Acid Content: Influence of Non-Microbial Biostimulants

The determination of organic acid content are crucial aspects in the overall quality assessment of a given food. The composition of the organic acid profile in both fruiting and leafy vegetables influences not only flavor but also shelf life, nutraceutical value, and acceptability [57]. Analyses by ion chromatography of arugula plant tissues showed that malate and citrate accounted for almost all of the organic acid content with comparable average values (21.6 and 20.2 g kg^−1^ dw, respectively) (Table 5). Although oxalate content was enormously lower (on average 1.08 g kg^−1^ dw) compared to what was recorded for malate and citrate, its deleterious effect on dietary calcium bioavailability and in subsequent kidney stone formation is widely documented in the literature [58]. Even though oxalate has low toxicity in humans, its abundant presence in some leafy vegetables is an important qualitative discriminator.

Considering the involvement of organic acids in different biochemical pathways, this result is not at all surprising. Interestingly, in addition to being important photosynthetic intermediates, organic acids in plants by altering cellular pH can indirectly influence the biosynthesis of phenolic compounds [59]. The increase in total flavonol and phenolic acid content recorded in SWE- and V-PH-treated arugula plants, respectively, could be related to a significant reduction in malate content. Not surprisingly, cytosolic proteins involved in organic acid metabolism by coordinating malate concentrations in response to stressors [60] might have been activated by the application of SWE and V-PH biostimulants. On the other hand, with regard to oxalate content, the foliar application of the tested biostimulants, regardless of their origin, resulted in a significant reduction (by 32.6% on average) compared to the control, making arugula consumption even “safer”.

## 4. Conclusions

Although the results of the previous experiment highlighted how foliar application of natural products with biostimulant action is a viable and practical strategy for increasing the organic yield of arugula [28], in this study, we demonstrated how the application of the same biostimulants (vegetable-derived protein hydrolysate, plant extract, and seaweed extract) positively influenced the biosynthesis of key secondary metabolites. From the results reported, it is clear how the different origin and composition of the investigated products influence the biosynthesis of distinct metabolic classes. Specifically, while the foliar application of the biostimulant product obtained by enzymatic hydrolysis of vegetables improved the content of phenolic acids and glucosinolates, the application of the extract obtained from seaweed positively regulated the production of flavonoids. In light of this, a greater understanding of how the numerous and still poorly understood bioactive molecules in non-microbial biostimulant formulations could guide producers to obtain agricultural products characterized by superior nutraceutical power, thus meeting consumer demands.

## Figures and Tables

**Table 1:** Chemical properties of glucosinolate compounds analyzed by mass spectrometry.

Compounds	Glucosinolate Classification	Adduct Ion	Chemical Formula	Theoretical Mass (*m/z*)	Measured Mass (*m/z*)	Accuracy	LOD (mg kg^−1^)	LOQ (mg kg^−1^)
						(Δ mg kg^−1^)		
Glucotropaeolin *	Aromatic	[M-H]^−^	C_14_H_19_NO_9_S_2_	408.04285	408.04269	−0.39212	0.052	0.156
Sinigrin *	Aliphatic	[M-H]^−^	C_10_H_17_NO_9_S_2_	358.02719	358.02747	0.78206	0.052	0.156
Glucoraphenin	Aliphatic	[M-H]^−^	C_12_H_21_NO_10_S_3_	434.02548	434.04515	45.31992	-	-
Glucoraphanin	Aliphatic	[M-H]^−^	C_12_H_23_NO_10_S_3_	436.04113	436.04095	−0.41281	-	-
Glucoiberin	Aliphatic	[M-H]^−^	C_11_H_21_NO_10_S_3_	422.02548	422.02515	−0.78194	-	-
Progoitrin	Aliphatic	[M-H]^−^	C_11_H_19_NO_10_S_2_	388.03776	388.03751	−0.64427	-	-
Sinalbin	Aliphatic	[M-H]^−^	C_14_H_19_NO_10_S_2_	424.03776	424.03778	0.04717	-	-
Glucoerucin	Aliphatic	[M-H]^−^	C_12_H_23_NO_9_S_3_	420.04622	420.04599	−0.54756	-	-
Glucobrassicin	Indole	[M-H]^−^	C_16_H_20_N_2_O_9_S_2_	447.05374	447.05389	0.33553	-	-
Gluconasturtiin	Aromatic	[M-H]^−^	C_15_H_21_NO_9_S_2_	422.05850	422.05960	2.60627	-	-
Glucoberteroin	Aliphatic	[M-H]^−^	C_13_H_25_NO_9_S_3_	434.06187	434.06198	0.25342	-	-
Glucobarbarin	Aliphatic	[M-H]^−^	C_15_H_21_NO_10_S_2_	438.05341	438.04999	−7.80727	-	-
Glucocheirolin	Aliphatic	[M-H]^−^	C_11_H_21_NO_11_S_3_	438.02039	438.01996	−0.98169	-	-
Glucoalyssin	Aliphatic	[M-H]^−^	C_13_H_25_NO_10_S_3_	450.05678	450.05704	0.57770	-	-

* Compounds in bold were quantified using the real standard.

**Table 2:** Effect of different non-microbial biostimulants on the content of glucosinolates (glucosinalbin, glucoerucin, glucobrassicin, gluconasturtin, glucoalyssin, glucoraphenin, glucoiberin, progoitrin, glucoraphanin, and glucocheirolin) of greenhouse-grown wild arugula.

Treatment	Glucosinalbin	Glucoerucin	Glucobrassicin	Gluconasturtin	Glucoalyssin	Glucoraphenin	Glucoiberin	Progoitrin	Glucoraphanin	Glucocheirolin
	µg g^−1^ DW									
Control	7.2 ± 0.213	74.7 ± 2.75 b	4.23 ± 0.09	3.65 ± 0.03	3.25 ± 0.10	-	2.72 ± 0.17 ab	12.17 ± 1.02 a	202.25 ± 1.73 ab	5.27 ± 0.33 b
PE	6.95 ± 0.159	85.7 ± 1.97 a	4.08 ± 0.16	3.67 ± 0.01	2.97 ± 0.20	-	2.51 ± 0.07 b	8.43 ± 0.52 b	212.48 ± 3.99 a	4.15 ± 0.18 b
V-PH	6.58 ± 0.233	91.83 ± 2.1 a	4.21 ± 0.23	3.73 ± 0.06	2.75 ± 0.40	1.82 ± 0.01	3.17 ± 0.17 ab	10.87 ± 0.71 ab	197.45 ± 2.34 b	9.32 ± 0.47 a
SWE	7.17 ± 0.142	85.78 ± 2.5 a	4.72 ± 0.23	3.95 ± 0.17	3.09 ± 0.16	1.77 ± 0.01	3.77 ± 0.48 a	11.7 ± 1.07 ab	191.77 ± 1.95 b	8.67 ± 0.27 a
	n.s	**	n.s	n.s	n.s	n.s	*	*	***	***

Different letters indicate significant mean differences. ns, *, **, and *** denote non-significant or significant effects at *p* ≤ 0.05, 0.01, and 0.001, respectively.

**Table 3:** Chemical properties of polyphenols compounds analyzed by mass spectrometry.

Compounds	Adduct Ion	Chemical Formula	Theoretical Mass (*m/z*)	Measured Mass (*m/z*)	Accuracy (Δ mg kg^−1^)	LOD (mg kg^−1^)	LOQ (mg kg^−1^)
Quinic acid *	[M-H]^−^	C_7_H_12_O_6_	191.05531	191.05611	4.18727	0.013	0.039
Protocatechuic acid *	[M-H]^−^	C_7_H_6_O_4_	153.01930	153.01857	−4.77064	0.013	0.039
Chlorogenic acid *	[M-H]^−^	C_16_H_18_O_9_	353.08780	353.08798	0.50979	0.013	0.039
Caffeic acid *	[M-H]^−^	C_9_H_8_O_4_	179.03498	179.03455	−2.40177	0.013	0.039
*p*-Coumaric acid *	[M-H]^−^	C_9_H_8_O_3_	163.04001	163.03937	−3.92542	0.013	0.039
Genistein *	[M-H]^−^	C_15_H_10_O_5_	269.04554	269.04562	0.29735	0.013	0.039
Ferulic acid *	[M-H]^−^	C_10_H_10_O_4_	193.05063	193.05016	−2.43459	0.026	0.078
Naringin *	[M-H]^−^	C_27_H_32_O_14_	579.17193	579.17212	0.32805	0.013	0.-039
Rutin *	[M-H]^−^	C_27_H_30_O_16_	609.14611	609.14673	1.01782	0.013	0.039
Myricitrin *	[M-H]^−^	C_21_H_20_O_12_	463.08820	463.08701	−2.56970	0.013	0.039
Quercetin *	[M-H]^−^	C_15_H_10_O_7_	301.03538	301.03508	−0.29735	0.019	0.057
Kaempferol *	[M-H]^−^	C_15_H_10_O_6_	285.04046	285.04086	1.40330	0.013	0.039
Apigenin *	[M-H]^−^	C_15_H_10_O_5_	269.04555	269.04572	0.63190	0.013	0.039
Luteolin *	[M-H]^−^	C_15_H_10_O_6_	285.04046	285.04086	1.40331	0.026	0.078
Quercetin 3-galattoside *	[M-H]^−^	C_21_H_20_O_12_	463.08820	463.08884	1.38203	0.026	0.078
Quercetin-3-o-feruloyyl-sophoros	[M-H]^−^	C_37_H_38_O_20_	801.18836	801.18781	−0.68648	-	-

* Compounds in bold were quantified using the real standard.

**Table 4:** Effect of different non-microbial biostimulants on phenolic acid and flavonoid profile of greenhouse-grown wild arugula.

Treatment	Chlorogenic Acid	Caffeic Acid	Ferulic Acid	Myricitrin	Rutin	Quercetin 3 Galactoside	Quercetin-3-o-Feruloyyl-Sophoros
	mg g^−1^ DW						
Control	2.08 ± 0.07 a	85.74 ± 1.76 ab	10.78 ± 0.65 c	11.79 ± 0.66 b	81.07 ± 2.03 a	12.35 ± 1.28 b	47.11 ± 0.83 a
PE	1.35 ± 0.14 b	76.02 ± 1.35 c	15.55 ± 0.70 b	7.62 ± 1.15 c	65.89 ± 1.60 b	8.92 ± 0.61 b	51.66 ± 1.46 a
V-PH	0.98 ± 0.07 b	90.67 ± 3.71 a	20.92 ± 0.9 a	3.37 ± 0.83 d	70.04 ± 0.35 b	4.29 ± 0.84 c	37.08 ± 2.62 b
SWE	2.11 ± 0.06 a	79.71 ± 0.89 bc	17.29 ± 0.69 b	17.12 ± 0.98 a	86.55 ± 1.39 a	17.22 ± 0.29 a	52.14 ± 1.38 a
	***	**	***	***	***	***	***

Different letters indicate significant mean differences. ** and ***: *p* ≤ 0.01 and 0.001, respectively.

**Table 5:** Effect of different non-microbial biostimulants on organic acid content (malate, oxalate, and citrate) of greenhouse-grown wild arugula.

Treatment	Malate	Oxalate	Citrate
	g kg^−1^ DW		
Control	23.66 ± 0.91 a	1.43 ± 0.01 a	19.72 ± 1.11 ab
PE	23.39 ± 0.27 a	0.99 ± 0.07 b	20.69 ± 0.25 ab
V-PH	19.39 ± 0.27 b	0.94 ± 0.03 b	22.53 ± 0.67 a
SWE	20.09 ± 0.85 b	0.96 ± 0.07 b	17.87 ± 0.25 b
	***	***	**

Different letters indicate significant mean differences. ** and ***: *p* ≤ 0.01 and 0.001, respectively.

## Data Availability

The original contributions presented in the study are included in the article/Supplementary Material (Table S2).

## Supplementary Materials

The following supporting information can be downloaded at https://www.mdpi.com/article/10.3390/foods14142489/s1. Supplementary Table S1: Composition and chemical characteristics of nonmicrobial biostimulants used; Supplementary Table S2: All experimental data subjected to the analysis of variance (ANOVA).

## References

[1] Galanakis C.M., Rizou M., Aldawoud T.M., Ucak I., Rowan N.J. (2021). Innovations and technology disruptions in the food sector within the COVID-19 pandemic and post-lockdown era. Trends Food Sci. Technol..

[2] Bodirsky B.L., Dietrich J.P., Martinelli E., Stenstad A., Pradhan P., Gabrysch S., Mishra A., Weindl I., Le Mouël C., Rolinski S. (2020). The ongoing nutrition transition thwarts long-term targets for food security, public health and environmental protection. Sci. Rep..

[3] Kris-Etherton P.M., Petersen K.S., Després J.-P., Anderson C.A., Deedwania P., Furie K.L., Lear S., Lichtenstein A.H., Lobelo F., Morris P.B. (2021). Strategies for promotion of a healthy lifestyle in clinical settings: Pillars of ideal cardiovascular health: A science advisory from the American Heart Association. Circulation.

[4] Rasines L., Morera S., San Miguel G., Aguayo E. (2024). Exploring the total cost of whole fresh, fresh-cut and pre-cooked vegetables. Int. J. Life Cycle Assess..

[5] Caruso G., Parrella G., Giorgini M., Nicoletti R. (2018). Crop systems, quality and protection of *Diplotaxis tenuifolia*. Agriculture.

[6] Giordano M., El-Nakhel C., Caruso G., Cozzolino E., De Pascale S., Kyriacou M.C., Colla G., Rouphael Y. (2020). Stand-alone and combinatorial effects of plant-based biostimulants on the production and leaf quality of perennial wall rocket. Plants.

[7] Rana M.K. (2017). Vegetable Crop Science.

[8] Renna M., Montesano F.F., Serio F., Gonnella M. (2021). The Mediterranean diet between traditional foods and human health through culinary examples. Gastronomy and Food Science.

[9] Motti R., Bonanomi G., Lanzotti V., Sacchi R. (2020). The contribution of wild edible plants to the Mediterranean Diet: An ethnobotanical case study along the coast of Campania (Southern Italy). Econ. Bot..

[10] Brender J.D. (2020). Human health effects of exposure to nitrate, nitrite, and nitrogen dioxide. Just Enough Nitrogen: Perspectives on How to Get There for Regions with Too Much and Too Little Nitrogen.

[11] Karwowska M., Kononiuk A. (2020). Nitrates/nitrites in food—Risk for nitrosative stress and benefits. Antioxidants.

[12] Orouji N., Asl S.K., Taghipour Z., Habtemariam S., Nabavi S.M., Rahimi R. (2023). Glucosinolates in cancer prevention and treatment: Experimental and clinical evidence. Med. Oncol..

[13] Gasmi A., Gasmi Benahmed A., Shanaida M., Chirumbolo S., Menzel A., Anzar W., Arshad M., Cruz-Martins N., Lysiuk R., Beley N. (2024). Anticancer activity of broccoli, its organosulfur and polyphenolic compounds. Crit. Rev. Food Sci. Nutr..

[14] Pasini F., Verardo V., Caboni M.F., D’Antuono L.F. (2012). Determination of glucosinolates and phenolic compounds in rocket salad by HPLC-DAD–MS: Evaluation of *Eruca sativa* Mill. and *Diplotaxis tenuifolia* L. genetic resources. Food Chem..

[15] Ketnawa S., Reginio Jr F.C., Thuengtung S., Ogawa Y. (2022). Changes in bioactive compounds and antioxidant activity of plant-based foods by gastrointestinal digestion: A review. Crit. Rev. Food Sci. Nutr..

[16] Sharif M., Ejaz M., Nawaz A., Saeeda U.H., Naeem S., Khan S. (2024). Impact of Dietary Flavonoid Metabolism on Gut Microbiome: A Key Therapeutic Approach for the Management of Type 2 Diabetes Mellitus. Role of Flavonoids in Chronic Metabolic Diseases: From Bench to Clinic.

[17] Singh S., Singh R., Thakur P., Kumar R. (2018). Phytochemicals, functionality and breeding for enrichment of cole vegetables (*Brassica oleracea* L.). Phytochemicals in Vegetables: A Valuable Source of Bioactive Compounds.

[18] Khare S., Singh N., Singh A., Hussain I., Niharika K., Yadav V., Bano C., Yadav R.K., Amist N. (2020). Plant secondary metabolites synthesis and their regulations under biotic and abiotic constraints. J. Plant Biol..

[19] Aguirre-Becerra H., Vazquez-Hernandez M.C., Saenz de la O D., Alvarado-Mariana A., Guevara-Gonzalez R.G., Garcia-Trejo J.F., Feregrino-Perez A.A. (2021). Role of stress and defense in plant secondary metabolites production. Bioactive Natural Products for Pharmaceutical Applications.

[20] Nephali L., Piater L.A., Dubery I.A., Patterson V., Huyser J., Burgess K., Tugizimana F. (2020). Biostimulants for plant growth and mitigation of abiotic stresses: A metabolomics perspective. Metabolites.

[21] Ramakrishnan B., Maddela N.R., Venkateswarlu K., Megharaj M. (2021). Organic farming: Does it contribute to contaminant-free produce and ensure food safety?. Sci. Total Environ..

[22] Soltaniband V., Brégard A., Gaudreau L., Dorais M. (2022). Biostimulants promote plant development, crop productivity, and fruit quality of protected strawberries. Agronomy.

[23] Quintarelli V., Borgatti D., Baretta M., Stazi S.R., Allevato E., Pancaldi S., Baldisserotto C., Mancinelli R., Tedeschi P., Radicetti E. (2025). Microbial biofertilizers and algae-based biostimulant affect fruit yield characteristics of organic processing tomato. J. Sci. Food Agric..

[24] Farruggia D., Tortorici N., Iacuzzi N., Alaimo F., Leto C., Tuttolomondo T. (2024). Biostimulants improve plant performance of rosemary growth in agricultural organic system. Agronomy.

[25] Gavelienė V., Šocik B., Jankovska-Bortkevič E., Jurkonienė S. (2021). Plant microbial biostimulants as a promising tool to enhance the productivity and quality of carrot root crops. Microorganisms.

[26] Ganugi P., Fiorini A., Tabaglio V., Capra F., Zengin G., Bonini P., Caffi T., Puglisi E., Trevisan M., Lucini L. (2023). The functional profile and antioxidant capacity of tomato fruits are modulated by the interaction between microbial biostimulants, soil properties, and soil nitrogen status. Antioxidants.

[27] Li J., Lardon R., Mangelinckx S., Geelen D. (2024). A practical guide to the discovery of biomolecules with biostimulant activity. J. Exp. Bot..

[28] Ciriello M., Campana E., Colla G., Rouphael Y. (2024). An Appraisal of Nonmicrobial Biostimulants’ Impact on the Productivity and Mineral Content of Wild Rocket (*Diplotaxis tenuifolia* (L.) DC.) Cultivated under Organic Conditions. Plants.

[29] Colla G., Cardarelli M., Bonini P., Rouphael Y. (2017). Foliar applications of protein hydrolysate, plant and seaweed extracts increase yield but differentially modulate fruit quality of greenhouse tomato. HortScience.

[30] Formisano L., Ciriello M., El-Nakhel C., De Pascale S., Rouphael Y. (2021). Dataset on the effects of anti-insect nets of different porosity on mineral and organic acids profile of Cucurbita pepo L. fruits and leaves. Data.

[31] Izzo L., Castaldo L., Lombardi S., Gaspari A., Grosso M., Ritieni A. (2022). Bioaccessibility and antioxidant capacity of bioactive compounds from various typologies of canned tomatoes. Front. Nutr..

[32] Santamaria P., Elia A., Serio F. (2002). Effect of solution nitrogen concentration on yield, leaf element content, and water and nitrogen use efficiency of three hydroponically-grown rocket salad genotypes. J. Plant Nutr..

[33] Weightman R., Huckle A., Roques S., Ginsburg D., Dyer C. (2012). Factors influencing tissue nitrate concentration in field-grown wild rocket (Diplotaxis tenuifolia) in southern England. Food Addit. Contam. Part A.

[34] Bell L., Wagstaff C. (2019). Rocket science: A review of phytochemical & health-related research in Eruca & Diplotaxis species. Food Chem. X.

[35] Jonvik K.L., Nyakayiru J., Pinckaers P.J., Senden J.M., van Loon L.J., Verdijk L.B. (2016). Nitrate-rich vegetables increase plasma nitrate and nitrite concentrations and lower blood pressure in healthy adults. J. Nutr..

[36] Htwe N.M.P.S., Ruangrak E. (2021). A review of sensing, uptake, and environmental factors influencing nitrate accumulation in crops. J. Plant Nutr..

[37] Raffo A., Masci M., Moneta E., Nicoli S., Del Pulgar J.S., Paoletti F. (2018). Characterization of volatiles and identification of odor-active compounds of rocket leaves. Food Chem..

[38] Bell L., Wagstaff C. (2014). Glucosinolates, myrosinase hydrolysis products, and flavonols found in rocket (*Eruca sativa* and *Diplotaxis tenuifolia*). J. Agric. Food Chem..

[39] Kamal M.M., Akter S., Lin C.-N., Nazzal S. (2020). Sulforaphane as an anticancer molecule: Mechanisms of action, synergistic effects, enhancement of drug safety, and delivery systems. Arch. Pharmacal Res..

[40] Xu L., Nagata N., Ota T. (2018). Glucoraphanin: A broccoli sprout extract that ameliorates obesity-induced inflammation and insulin resistance. Adipocyte.

[41] Wagner A.E., Sturm C., Piegholdt S., Wolf I.M., Esatbeyoglu T., De Nicola G.R., Iori R., Rimbach G. (2015). Myrosinase-treated glucoerucin is a potent inducer of the Nrf2 target gene heme oxygenase 1—Studies in cultured HT-29 cells and mice. J. Nutr. Biochem..

[42] Genah S., Ciccone V., Filippelli A., Simonis V., Martelli A., Piragine E., Pagnotta E., Pecchioni N., Calderone V., Morbidelli L. (2024). Erucin, a natural isothiocyanate, exerts pro-angiogenic properties in cultured endothelial cells and reverts angiogenic impairment induced by high glucose. Phytother. Res..

[43] Tran T.L.C., Callahan D.L., Islam M.T., Wang Y., Arioli T., Cahill D. (2023). Comparative metabolomic profiling of Arabidopsis thaliana roots and leaves reveals complex response mechanisms induced by a seaweed extract. Front. Plant Sci..

[44] Martínez-Lorente S.E., Martí-Guillén J.M., Pedreño M.Á., Almagro L., Sabater-Jara A.B. (2024). Higher plant-derived biostimulants: Mechanisms of action and their role in mitigating plant abiotic stress. Antioxidants.

[45] Lephatsi M., Nephali L., Meyer V., Piater L.A., Buthelezi N., Dubery I.A., Opperman H., Brand M., Huyser J., Tugizimana F. (2022). Molecular mechanisms associated with microbial biostimulant-mediated growth enhancement, priming and drought stress tolerance in maize plants. Sci. Rep..

[46] Bell L., Oruna-Concha M.J., Wagstaff C. (2015). Identification and quantification of glucosinolate and flavonol compounds in rocket salad (*Eruca sativa*, *Eruca vesicaria* and *Diplotaxis tenuifolia*) by LC–MS: Highlighting the potential for improving nutritional value of rocket crops. Food Chem..

[47] Manach C., Scalbert A., Morand C., Rémésy C., Jiménez L. (2004). Polyphenols: Food sources and bioavailability. Am. J. Clin. Nutr..

[48] Shen N., Wang T., Gan Q., Liu S., Wang L., Jin B. (2022). Plant flavonoids: Classification, distribution, biosynthesis, and antioxidant activity. Food Chem..

[49] Tao Y., Zhang H., Wang Y. (2023). Revealing and predicting the relationship between the molecular structure and antioxidant activity of flavonoids. LWT.

[50] Zheng Y.-Z., Deng G., Liang Q., Chen D.-F., Guo R., Lai R.-C. (2017). Antioxidant activity of quercetin and its glucosides from propolis: A theoretical study. Sci. Rep..

[51] Olszowy-Tomczyk M., Wianowska D. (2023). Antioxidant properties of selected flavonoids in binary mixtures—Considerations on myricetin, kaempferol and quercetin. Int. J. Mol. Sci..

[52] Flores P., Pedreño M., Almagro L., Hernández V., Fenoll J., Hellín P. (2021). Increasing nutritional value of broccoli with seaweed extract and trilinolein. J. Food Compos. Anal..

[53] Ali O., Ramsubhag A., Jayaraman J. (2019). Biostimulatory activities of Ascophyllum nodosum extract in tomato and sweet pepper crops in a tropical environment. PLoS ONE.

[54] Ciriello M., Fusco G.M., Woodrow P., Carillo P., Rouphael Y. (2024). Unravelling the nexus of plant response to non-microbial biostimulants under stress conditions. Plant Stress.

[55] Rashmi H.B., Negi P.S. (2020). Phenolic acids from vegetables: A review on processing stability and health benefits. Food Res. Int..

[56] Bourne L.C., Rice-Evans C. (1998). Bioavailability of ferulic acid. Biochem. Biophys. Res. Commun..

[57] Pannico A., Kyriacou M.C., El-Nakhel C., Graziani G., Carillo P., Corrado G., Ritieni A., Rouphael Y., De Pascale S. (2022). Hemp microgreens as an innovative functional food: Variation in the organic acids, amino acids, polyphenols, and cannabinoids composition of six hemp cultivars. Food Res. Int..

[58] Mitchell T., Kumar P., Reddy T., Wood K.D., Knight J., Assimos D.G., Holmes R.P. (2019). Dietary oxalate and kidney stone formation. Am. J. Physiol.-Ren. Physiol..

[59] Flores P., Hellín P., Fenoll J. (2012). Determination of organic acids in fruits and vegetables by liquid chromatography with tandem-mass spectrometry. Food Chem..

[60] Bell L., Chadwick M., Puranik M., Tudor R., Methven L., Wagstaff C. (2022). Quantitative trait loci analysis of glucosinolate, sugar, and organic acid concentrations in Eruca vesicaria subsp. sativa. Mol. Hortic..

