# Supplementary material for: Differential Effects of Non-Microbial Biostimulants on Secondary Metabolites and Nitrate Content in Organic Arugula Leaves
Source: Foods. 2025 Jul 16;14(14):2489. doi: 10.3390/foods14142489 (PMC12294971; doi:10.3390/foods14142489)
Supplement: Supplementary file 1 [file foods-14-02489-s001.zip › Supplementary_foods-3711207_R1.pdf]

Supplementary Table S1: Composition and chemical characteristics of nonmicrobial biostimulants used.

	**Vegetable-Protein Hydrolysate (V-PH)**	**Plant Extract (PE)**	**Seaweed Extract (SWE)**
Free amino acids and soluble peptides	310 g kg^{-1}	51.9 g kg^{-1}	NA
$N-NO_3$	3.13 µg g^{-1} f.w	NA	NA
$N-NH_4$	6.00 µg g^{-1} f.w	NA	NA
Auxins	ND	1.81 mg kg^{-1}	NA
Cytokinins	ND	0.29 mg kg^{-1}	NA
Organic Carbon	NA	NA	0.2%
Mannitol	ND	ND	0.7 g L^{-1}

ND = not detected and NA= not available.
